# Sequence Variation in *DDAH1* and *DDAH2* Genes Is Strongly and Additively Associated with Serum ADMA Concentrations in Individuals with Type 2 Diabetes

**DOI:** 10.1371/journal.pone.0009462

**Published:** 2010-03-01

**Authors:** Sotoodeh Abhary, Kathryn P. Burdon, Abraham Kuot, Shahrbanou Javadiyan, Malcolm J. Whiting, Nicholas Kasmeridis, Nikolai Petrovsky, Jamie E. Craig

**Affiliations:** 1 Department of Ophthalmology, Flinders Medical Centre and Flinders University, Adelaide, South Australia, Australia; 2 Department of Endocrinology, Flinders Medical Centre and Flinders University, Adelaide, South Australia, Australia; 3 Chemical Pathology Laboratory, SA Pathology, Adelaide, South Australia, Australia; University of Bremen, Germany

## Abstract

**Background:**

Asymmetric dimethylarginine (ADMA), present in human serum, is an endogenous inhibitor of nitric oxide synthase and contributes to vascular disease. Dimethylarginine dimethylaminohydrolase (DDAH) is an ADMA degrading enzyme that has two isoforms: DDAHI and DDAHII. We sought to determine whether serum ADMA levels in type 2 diabetes are influenced by common polymorphisms in the *DDAH1* and *DDAH2* genes.

**Methodology/Principal Findings:**

Relevant clinical parameters were measured and peripheral whole blood obtained for serum and genetic analysis on 343 participants with type 2 diabetes. Serum ADMA concentrations were determined by mass spectroscopy. Twenty six tag SNPs in the *DDAH1* and 10 in the *DDAH2* gene were genotyped in all subjects and tested for association with serum ADMA levels. Several SNPs and haplotypes in the *DDAH* genes were strongly associated with ADMA levels. Most significantly in the *DDAH1* gene, rs669173 (p = 2.96×10^−7^), rs7521189 (p = 6.40×10^−7^), rs2474123 (p = 0.00082) and rs13373844 (p = 0.00027), and in the *DDAH2* gene, rs3131383 (p = 0.0029) and the TGCCCAGGAG haplotype (p = 0.0012) were significantly associated with ADMA levels. Sub-analysis by diabetic retinopathy (DR) status revealed these variants were associated with ADMA levels predominantly in participants without DR. Combined analysis of the most strongly associated SNPs in *DDAH1* (rs669173) and *DDAH2* (rs3131383) revealed an additive effect (p = 1.37×10^−8^) on ADMA levels.

**Conclusions/Significance:**

Genetic variation in the *DDAH1* and *2* genes is significantly associated with serum ADMA levels. Further studies are required to determine the pathophysiological significance of elevated serum ADMA in type 2 diabetes and to better understand how *DDAH* gene variation influences ADMA levels.

## Introduction

Asymmetric dimethylarginine (ADMA) is an endogenous inhibitor of nitric oxide synthase (NOS), the key endothelial enzyme that converts L-arginine to L-citrulline and nitric oxide (NO). Endothelium-derived NO helps to maintain vascular homeostasis through vasodilatation, suppression of inflammation and inhibition of the proliferation of vascular smooth muscle cells [1], [2], platelet adhesion and aggregation [3], [4].

ADMA levels have been shown to be increased in individuals with diabetes mellitus [5]. Endothelial dysfunction, such as occurs in hyperglycemia, is associated with decreased NOS activity and NO bioavailability resulting in vasoconstriction and increased reactive oxygen species. This leads to impaired ocular hemodynamics [6]. Recently, elevated serum ADMA in both diabetes [5] and its complications, including retinopathy [7], [8], [9], [10] and nephropathy [10], [11], have been reported.

Dimethylarginine dimethylaminohydrolase (DDAH) is the enzyme responsible for the degradation of ADMA into citrulline and dimethylamine [12], [13]. Over 90% of endogenous ADMA is metabolised by DDAH with the remainder renally excreted [14]. DDAH is expressed as two isoforms encoded by different genes. DDAHI predominates in tissues expressing neuronal NOS (nNOS), and is encoded by the *DDAH1* (OMIM#604743) gene on chromosome 1p22. DDAHII is found in highly vascular tissues expressing endothelial NOS (eNOS) [15], [16], and immune tissues expressing inducible NOS (iNOS) [16], and is encoded by the *DDAH2* (OMIM#604744) gene on chromosome 6p21.3.

Evidence for the metabolic control of ADMA by DDAH genes, and their influence on endothelial cells has been provided by animal studies. *DDAH1* overexpressing transgenic mice had a two fold reduction in plasma ADMA associated with a 2 fold increase in tissue NOS activity [17]. Conversely, *DDAH1* knockout mice had increased pulmonary endothelial permeability as a result of ADMA elevation, which was prevented by overexpression of *DDAH1* and *DDAH2* in endothelial cells [18].

We aimed to determine whether serum ADMA levels are influenced by common single nucleotide polymorphisms (SNPs) in *DDAH1* and *DDAH2* genes in a large Australian cohort of individuals with type 2 diabetes, and found that genetic variation in the *DDAH1* and *DDAH2* genes significantly and additively affects serum ADMA concentrations.

## Results

Three hundred and forty three participants with type 2 diabetes were included. Disease duration, smoking, nephropathy and diabetic retinopathy were significantly associated with serum ADMA levels in all participants (p<0.05, Table 1). These variables were subsequently adjusted for in multivariate analyses. Subjects with no retinopathy (n = 225) were more likely to be female, had shorter duration of disease, significantly lower HbA1c levels and less nephropathy when compared to subjects with blinding diabetic retinopathy (n = 118, data not shown).

**Table 1:** Clinical characteristics [number of subjects (%) or mean±standard deviation] of all participants and associations with serum ADMA levels.

Clinical characteristics	All participants (n = 343)	Pearson Correlation	P value
Female (%)	162 (47)	−0.054	0.316
Age (years)	63.7±12.9	0.006	0.914
Disease duration (years)	14.8±8.9	0.138	0.011
HbA1c (%)	7.9±1.8	0.099	0.098
BMI (kg/m2)	32.3±7.2	0.074	0.182
Hypercholesterolemia (%)	249 (70)	−0.019	0.728
Smoker (%)	178 (52)	0.115	0.033
Retinopathy (%)	118 (35)	0.115	0.034
Nephropathy (%)	95 (28)	0.111	0.041
GFR (mL/min)	121.5±407.4	0.022	0.702
Hypertension (%)	285 (83)	0.050	0.352

**Figure 1:**
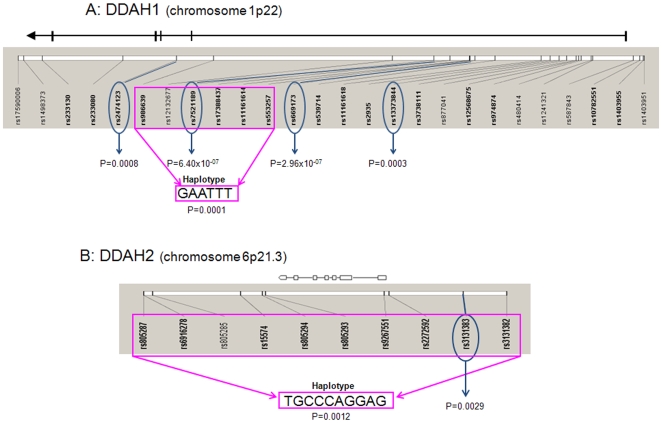
Idiogram of significantly associated SNPs and haplotypes of *DDAH I* (1A) and *DDAH* II (1B) with serum ADMA levels. Scales of genomic regions are estimates. P values are presented for significant SNPs and haplotype associations have been adjusted for disease duration, age, hypertension, smoking, nephropathy and diabetic retinopathy.

Several SNPs in the *DDAH* genes were significantly associated with serum ADMA levels after adjusting for associated variables in the multivariate analyses (Table 2 and Figure 1), with the most significant in *DDAH1* being rs669173 [p = 2.96×10^−7^ in the genotypic model, Beta coefficient (B): −0.03, 95% CI: −0.04 to −0.02], rs7521189 (p = 6.40×10^−7^ in the additive model, B: −0.03, 95% CI: −0.04 to −0.01), rs2474123 (p = 0.00082 in the additive model, B: 0.02, 95%CI: 0.01–0.04), rs13373844 (p = 0.00027 in the dominant model, B: −0.03, 95% CI: −0.05 to −0.02) and rs986639 (p = 0.0015 in the genotypic model, B: 0.03, 95% CI: 0.01–0.05). The SNPSpD method for multiple testing correction in SNP association studies estimated a total of 17 independent tests for *DDAH1* analyses and after correcting for multiple testing, the SNPs listed above remained significantly associated with serum ADMA levels (p<0.03, Table 2).

**Table 2:** Association of *DDAH* tag SNPs and with serum ADMA levels in all participants.

				Additive	Genotypic	Dominant	Recessive
	SNP #	SNP name	Minor allele	P value	P value	P value	P value
DDAH1	1	rs17590006	G	0.0509	**0.0109** †	**0.0037** †	0.1331
	2	rs1498373	T	**0.0122** †	**0.0171** †	**0.0129**	**0.0459**
	3	rs233130	G	**0.0067** †	**0.0082** †	**0.0063** †	**0.0320** †
	4	rs233080	A	0.4799	0.5185	0.6694	0.3679
	5	rs2474123	A	**0.0008** * †	**0.0024** †	**0.0032** †	**0.0095** †
	6	rs986639	C	**0.0020** * †	**0.0015** * †	**0.0093** †	**0.0034** †
	7	rs12132677	C	0.3125	0.2603	0.1188	0.3919
	8	rs7521189	A	**6.40E-07** * †	**3.08E-06** * †	**0.0073**	**2.29E-05** * †
	9	rs17388437	C	0.2117	0.4512	0.9184	0.2106
	10	rs11161614	G	0.1651	0.0799	0.0312	0.2791
	11	rs553257	C	0.2730	0.4311	0.2995	0.3152
	12	rs669173	C	**8.52E-07** * †	**2.96E-07** * †	**0.0006** * †	**8.48E-06** * †
	13	rs539714	C	**0.0397**	0.0957	0.3206	**0.0423**
	14	rs11161618	T	0.1986	0.3762	0.1887	0.3788
	15	rs2935	A	**0.0402** †	**0.0354** †	**0.0448**	**0.0457** †
	16	rs13373844	C	**0.0065** †	**0.0007** †	**0.0003** * †	**0.0447**
	17	rs3738111	C	0.4493	0.4404	0.4608	0.4253
	18	rs877041	A	0.3845	0.5145	0.2565	0.6132
	19	rs12568675	C	**0.0080** †	**0.0086** †	**0.0223**	**0.0102** †
	20	rs974874	C	0.2716	0.5220	0.4315	0.3135
	21	rs480414	A	0.7295	0.9286	0.7453	0.7703
	22	rs1241321	C	0.2872	0.3422	0.1526	0.4686
	23	rs587843	C	0.9023	0.7975	0.5345	0.9364
	24	rs10782551	A	0.9029	0.1900	0.0844	0.8544
	25	rs1403955	C	0.1959	0.3157	0.1524	0.3582
	26	rs1403951	T	0.3152	0.4972	0.2431	0.5851
DDAH2	1	rs805287	C	0.4247	0.7155	0.5545	0.4719
	2	rs6916278	A	0.9751	0.9945	0.9164	0.9786
	3	rs805285	G	0.1883	0.4166	0.5521	0.1905
	4	rs15574	T	0.2355	0.3790	0.9151	0.1986
	5	rs805294	C	0.4001	0.6263	0.3565	0.5493
	6	rs805293	T	0.7413	0.9147	0.9231	0.6761
	7	rs9267551	C	0.6374	0.5282	0.2590	0.6907
	8	rs2272592	A	0.2848	0.4027	0.2462	0.3306
	9	rs3131383	A	**0.0179** †	**0.0038** * †	**0.0029** * †	**0.0272** †
	10	rs3131382	A	0.5141	0.8071	0.7988	0.5127

Note: p values have been adjusted for duration of diabetes, age, hypertension, smoking, nephropathy and retinopathy status. Results are shown only if genotypes were carried by 5 or more participants. Significant p values are shown in bold type.

*  =  p-values survive correction for multiple testing.

†  =  p values are also significant in no diabetic retinopathy subset.

SNP rs3131383 in *DDAH2* was significantly associated with serum ADMA levels (p = 0.0029 in the dominant model, B: −0.03, 95% CI: −0.06 to −0.01, Table 2 and Figure 1). Seven independent tests for *DDAH2* were estimated by the SNPSpD method and after correcting for multiple testing, rs3131383 (p = 0.034 in the dominant model) remained significantly associated with ADMA levels in all participants (Table 2).

Sub-analysis by diabetic retinopathy status in the multivariate analyses revealed the majority of these SNPs to be associated with ADMA level only in the subgroup of participants that had no diabetic retinopathy, most significantly rs669173 (p = 4.66×10^−5^ in the additive model, B: −0.05, 95% CI: −0.08 to −0.03), rs2474123 (p = 0.00086 in the additive model, B: 0.04, 95% CI: 0.01–0.06) and rs7521189 (p = 0.0027 in the additive model, B: −0.03, −0.06 to −0.01, Table 2). These SNPs remained significant after correction for multiple testing. In the blinding diabetic retinopathy cases, rs669173 (p = 0.017 in the additive model), and rs7521189 (p = 0.023 in the additive model) were nominally significant, however did not survive correction for multiple testing (Table 2). Although association of rs3131383 in *DDAH2* with ADMA level was not statistically significantly associated in the smaller blinding retinopathy group, the direction of the association with ADMA was the same as that for no retinopathy controls and the full dataset (p = 0.143, B: −0.03 95% CI: −0.07–0.01).

To assess whether there are additive effects of polymorphisms in the two genes on serum ADMA, the total number of minor alleles of the most significantly associated SNP in each gene (rs669173 and rs3131383) was counted in each individual and included in the linear regression along with the clinical covariates. Multiple minor alleles were associated with lower serum ADMA (Supplementary Table S1B). The total number of minor alleles of the two SNPs was significantly associated with ADMA serum level after adjusting for relevant clinical variables (p = 1.37×10^−08^, B: −0.03, 95% CI: −0.04 to −0.02, Table 3). This model accounted for more of the variation (r^2^ = 0.147) than including each SNP in the regression as a separate factor with (r^2^ = 0.119) or without (r^2^ = 0.119) an interaction term between the two SNPs. Thus the two genes appear to influence ADMA levels additively.

**Table 3:** Association of the combination of the most significantly associated *DDAH1* (rs669173) and *DDAH2* (rs3131383) SNPs with serum ADMA levels in all participants.

Variable	B	Lower 95% CI	Upper 95% CI	P value
Combined DDAH1 and 2 SNPs	−0.03	−0.04	−0.02	1.37×10^−08^
Disease duration	0.00	0.00	0.00	0.006
Age	0.00	0.00	0.00	0.319
Hypertension	0.01	−0.01	0.03	0.431
Smoking	0.02	0.01	0.04	0.011
Nephropathy	0.01	0.00	0.03	0.144
DR	0.01	0.00	0.03	0.121
(Constant)	0.73	0.66	0.81	

B = beta coefficient.

For the haplotype analyses, observation of the *DDAH* SNPs in HapMap revealed three blocks of linkage disequilibrium in *DDAH1* and one block in *DDAH2* (Table 4). As block 2 of *DDAH1* consisted of 11 tag SNPs, it was further subdivided into two blocks for the haplotype analyses. After adjusting for associated variables in the multivariate analyses, several *DDAH1* haplotypes were significantly associated with ADMA levels, with the GAATTT haplotype of block 2A (p = 0.00012, B: −0.03, 95% CI: −0.04 to −0.01, Table 4 and Figure 1), and the TATAGTGGAG haplotype of block 3 (p = 0.00074, B: −0.02, 95% CI: −0.04 to −0.01) being the most significantly associated (Table 4). The TGCCCAGGAG of *DDAH2* was significantly associated with serum ADMA levels (p = 0.0012 B: −0.03 95% CI: −0.05 to −0.01, Table 4 and Figure 1). Seven haplotypes remained significant after Bonferroni correction (Table 4). Sub-analysis by diabetic retinopathy status revealed 5 of these haplotypes in *DDAH1* and the single associated haplotype in *DDAH2* to be significantly associated with serum ADMA level in the no diabetic retinopathy subgroup (Table 4) in the multivariate analyses. No haplotypes were associated with ADMA level in the blinding diabetic retinopathy subgroup after correction for multiple testing.

**Table 4:** Association of common *DDAH* haplotypes (>2% frequency) with ADMA levels.

*DDAH* gene	Haplotype block	Haplotype	Haplotype frequency	P value
1	Block 1	ACAGG	0.055	**0.0483**
	(SNPs 1–5)	ACAGA	0.091	0.2800
		GCAGG	0.236	**0.0027** * †
		ACAAG	0.287	0.9190
		ATGGA	0.326	**0.0023** * †
1	Block 2A	CAGTGT	0.034	0.1410
	(SNPs 6–11)	GAGCTT	0.035	0.6610
		GCGTGT	0.084	0.6920
		CCGTGT	0.098	**0.0044** *
		GAATTC	0.187	0.3250
		GAGTTT	0.256	**0.0139**
		GAATTT	0.279	**0.0001** * †
1	Block 2B	CTCGC	0.051	**0.0252**
	(SNPs 12–16)	TTCAA	0.091	**0.0203**
		TTCGA	0.124	0.0934
		TCCGA	0.144	0.1960
		CTCGA	0.149	0.0844
		TTTGA	0.193	0.0561
		CTTGC	0.224	**0.0030** * †
1	Block 3	TATAGTGGAG	0.276	**0.0007** * †
	(SNPs 17–26)	TACAGCGGAG	0.093	**0.0041** *
		TGTAACCAAG	0.084	0.0553
		TGTAGTCGAG	0.036	0.2330
		TGTAACCGCT	0.101	0.2830
		TGTCGTGGCT	0.260	0.5820
		TGTAACGGAG	0.027	0.8180
		CGTAATCGAT	0.080	0.9550
2	Block 1	CGCCTTGGCG	0.043	0.8070
	(All SNPs)	TGCCTACACA	0.052	0.3660
		TGCCTACACG	0.052	0.2750
		TGCCTAGACG	0.054	0.9140
		CGGCCAGGCG	0.064	0.3470
		TACCTTGGCG	0.067	0.9030
		TGCCCAGGAG	0.111	**0.0012** * †
		CGGTCAGGCG	0.199	0.9650
		TGCCTTGGCG	0.348	0.8960

Note: SNPs correspond to the order of those shown in Table 2. P values have been adjusted for duration of diabetes, age, hypertension, smoking, nephropathy and retinopathy status. Significant p values are shown in bold type.

*  =  p values survive correction for multiple testing.

†  =  p values are also significant in no diabetic retinopathy subset.

## Discussion

This study found genetic variation in the *DDAH1* and *DDAH2* genes to be significantly associated with serum ADMA levels in participants with type 2 diabetes. To our knowledge, this is the first study of its kind to investigate genetic variation in DDAH genes and their association with serum ADMA levels in patients with type 2 diabetes.

ADMA is an endogenous inhibitor of NOS and therefore NO bioavailability. The role of NO in the development of insulin resistance is supported by eNOS [19], [20] and nNOS [20] knockout mice studies. Similarly, previous studies have shown serum ADMA elevation to be significantly associated with diabetes [5] and insulin resistance [21] in humans. Animal studies have also supported the role of ADMA in insulin resistance, with DDAH expression shown to play a role [22]. When compared to wild type mice, in response to a glucose challenge, Sydow *et al* found DDAH1 transgenic mice to show a blunted increase in ADMA, plasma insulin and glucose [22]. Hyperglycemia has also been shown to lead to decreased DDAH activity and subsequent ADMA elevation [23]. A variety of other factors have also been shown to be involved in control of *DDAH* expression and activity. For example, oxidative stress and inflammation in cultured human endothelial cells decreases DDAH activity and upregulates ADMA synthesis [24], [25] subsequently leading to a reduction in NO synthesis [26]. Variation in the promoter region of *DDAH2* influences its expression [27]. Increased NO has also been shown to upregulate *DDAH2* expression in cultured rat aortic endothelial cells, indicating a possible positive feedback mechanism [28].

NO is also a key player in protection against microvascular damage [6], and serum ADMA levels have been found to be increased in conditions associated with endothelial dysfunction, including hypertension, hypercholesterolemia, hyperhomocysteinemia [29], [30] and diabetic retinopathy [7], [8], [9], [10]. Although DDAHI is primarily expressed in tissues producing nNOS [15], [16] and DDAHII in tissues producing eNOS and iNOS [16], all three isoforms of NOS have been isolated from the retina [31], [32], [33], [34]. Decreased retinal expression of eNOS [31] and increased expression of iNOS [32], [33] and nNOS [34] have been shown to be associated with hyperglycemia and diabetic retinopathy in both animal and human studies.

ADMA is present in the aqueous humor of the human eye. In a recent proteomic study with relatively small numbers, aqueous humor and serum ADMA levels were significantly higher in subjects with diabetes and those with severe diabetic retinopathy when compared to non-diabetic controls [9]. Sub-analysis by diabetic retinopathy status in this study revealed serum ADMA levels to be associated with common genetic variation in *DDAH* genes, primarily in our cohort of individuals with type 2 diabetes *without* diabetic retinopathy. However, for the mostly significantly associated SNPs, the serum ADMA levels trend in the same direction, and a true association may exist in the smaller retinopathy cohort which is less powered to detect the effect. The participants with no diabetic retinopathy had significantly lower ADMA levels compared to those with diabetic retinopathy [10]. Although the lack of highly significant association in the retinopathy group is likely attributable to power, it is possible that other influences play a greater role in determining serum ADMA levels once microvascular damage has occurred. Impairment of ADMA metabolism may occur due to oxidative stress and inflammation involved in endothelial dysfunction and microvascular damage. However, some studies have found elevated retinal NO in proliferative diabetic retinopathy [35], therefore ADMA elevation may actually be a compensatory mechanism to decrease pathologically elevated NO. It remains unclear whether ADMA elevation in diabetic retinopathy is a causal association or occurs as a result of endothelial dysfunction, and these potential mechanisms are not mutually exclusive.

Diabetic retinopathy is a debilitating complication of diabetes mellitus with a multifactorial pathogenesis and limited treatment options. Further prospective and functional studies investigating the pathophysiological significance of elevated serum ADMA in the development of diabetic retinopathy and other diabetes complications are required. Ultimately, if causal relationships are established, this could lead to design of future therapeutic or preventative strategies to correct NO levels in the ocular environment, thereby retarding or preventing the development of diabetic retinopathy.

Many of the most significantly associated *DDAH1* SNPs in this study are located in intron 1 of the gene. The significantly associated *DDAH2* SNP (rs3131383), although within the block of linkage disequilibrium that contains *DDAH2*, is actually within the chloride intracellular channel 1 (*CLIC1*) gene and 6255 bp from the *DDAH2* transcription start site. *CLIC1* encodes chloride ion channels that regulate fundamental cellular processes including transepithelial transport, maintenance of intracellular pH, and regulation of cell volume and cell cycle [36], [37], [38]. No associations have been reported between *CLIC1* and serum DDAH or ADMA. The associated SNPs in both genes could either tag functional SNPs that affect expression level (eg promoter or other regulatory variants), or coding variants that affect DDAH protein activity. Very few if any common coding variants have been reported in these genes and therefore deep resequencing may be required to determine if they do exist.

In conclusion, genetic variation in *DDAH1* and *DDAH2* genes was found to be strongly and significantly associated with serum ADMA levels in patients with type 2 diabetes, especially in those without retinopathy. Further studies are required to assess *DDAH* sequence variation and its influence on DDAH activity and serum ADMA levels both in individuals with and without diabetes to increase understanding of this complex pathway in normal and pathogenic conditions.

## Methods

### Ethics Statement

Ethics approval was obtained from the Human Research Ethics Committees of Flinders Medical Centre, Royal Adelaide Hospital and Queen Elizabeth Hospital in Adelaide, Australia, and written informed consent was obtained from all participants.

### Subject Recruitment

Subjects were recruited from ophthalmology and endocrinology outpatient clinics of three tertiary hospitals in metropolitan Adelaide, South Australia, initially to study the genetics of blinding diabetic retinopathy. Participants were over 18 years of age and were required to have type 2 diabetes of at least 5 years duration and be on oral hypoglycemic or insulin therapy.

A detailed questionnaire containing information regarding sex, age, ethnicity, age at diagnosis of diabetes, family diabetic history, co-existing risk factors, systemic complications of diabetes, ocular complications as a result of diabetic retinopathy, past ocular history, smoking history and alcohol intake was conducted in person for each participant. Renal function tests [serum creatinine, urine albumin and albumin∶creatinine ratio and glomerular filtration rate (GFR)], serum cholesterol and HbA1c levels were obtained from a state-wide database. Three recent HbA1c levels were averaged for each participant. For those cases diagnosed with blinding DR, HbA1c levels at the time of the ocular complication were used, and for controls with DM, HbA1c levels immediately prior to recruitment were averaged.

Peripheral whole blood was obtained from each participant and DNA extracted using the QiaAmp Blood Maxi Kit (Qiagen, Valencia, CA, USA). Blood pressure and body mass index (BMI) were measured in each participant. Patients were classified as hypertensive if they were on treatment for hypertension, or they had a blood pressure reading greater than or equal to 140/90 mmHg at the time of recruitment. Hypercholesterolemia was defined as total cholesterol of greater than 5.5 mmol/L, or current use of lipid lowering medication. Retinopathy status for the worst eye was clinically graded by an ophthalmologist according to the Early Treatment Diabetic Retinopathy Study criteria [39]. Participants were only included if they had either no retinopathy, or blinding retinopathy defined as severe non-proliferative diabetic retinopathy, proliferative diabetic retinopathy, or clinically significant macular edema. Nephropathy was defined as the presence of microalbuminuria (30–300 mg/day) or macroalbuminuria (>300 mg/day).

### *DDAH* SNP Selection and Genotyping

Using the tagger program implemented in Haploview 4.0 [40], tag single nucleotide polymorphisms (SNPs) across *DDAH1* and *DDAH2* genes, including the promoter region, were selected on the basis of linkage disequilibrium patterns observed in the Caucasian (CEU) samples genotyped as part of the International HapMap Project [41]. Only SNPs with minor allele frequency greater than 5% in HapMap were considered. Twenty six *DDAH1* and 10 *DDAH2* tag SNPs (Table 2) which captured all alleles with an r^2^ of at least 0.8 (mean r^2^ = 0.95), were genotyped in all individuals on the Sequenom iPLEX GOLD chemistry on an Autoflex Mass Spectrometer at the Australian Genome Research Facility, Brisbane, Australia.

### Measurement of Serum Concentrations of ADMA, SDMA and Arginine

Serum ADMA concentrations were determined in all participants [10] by liquid chromatography-tandem mass spectrometry of the butyl esters on an Applied Biosystems 3200 Q-Trap instrument (Applied Biosystems, Scoresby, Victoria), as described by Schwedhelm et al [42]. ®Deuterated internal standards (98 atom% ^2^H isotopic purity) were purchased from Cambridge Isotope Laboratories (Andover, MA) and were L-[^2^H_7_]-arginine for arginine quantitation and 2,3,3,4,4,5,5-[^2^H_7_]-ADMA for ADMA analyses. The between-run coefficient of variation for ADMA was determined as 4.0% at a concentration of 0.48 µmol/L.

### Statistical Analyses

Pearson correlation was undertaken for associations of clinical covariates with ADMA levels in SPSS (v15.0 SPSS Inc, Chicago, IL). Differences in clinical covariates between patients with and without diabetic retinopathy were calculated by Student's t-test or the chi-square test. ADMA levels were log transformed to approximate a normal distribution. Genotypic associations for all SNPs were assessed in PLINK (v1.06) [43]. Dominant, additive, recessive and genotypic models were considered with respect to the minor allele. Haplotypic analyses were performed in accordance with the observed linkage disequilibrium (LD) patterns in the Caucasian sample in HapMap. Multiple testing of individual SNPs was adjusted for using the Single Nucleotide Polymorphism Spectral Decomposition (SNPSpD) method of Nyholt [44] modified by Li and Ji [45] and Bonferroni correction was applied to haplotype analyses.

## Supporting Information

Table S1 (0.21 MB DOC)
