# Supplementary material for: Sequence Variation in DDAH1 and DDAH2 Genes Is Strongly and Additively Associated with Serum ADMA Concentrations in Individuals with Type 2 Diabetes
Source: PLoS One. 2010 Mar 1;5(3):e9462. doi: 10.1371/journal.pone.0009462 (PMC2830883; doi:10.1371/journal.pone.0009462)
Supplement: Table S1 — (0.21 MB DOC) [file pone.0009462.s001.doc]

Supplementary Table S1– Means of untransformed serum ADMA concentrations (μmol/L).

S1A: Means of untransformed serum ADMA concentrations (μmol/L) by genotype in all participants with T2DM.

| *DDAH* gene | SNP | Genotype | Genotype count | Genotype frequency | ADMA μmol/L (mean±SD) |
| --- | --- | --- | --- | --- | --- |
| DDAH1 | rs17590006 | AA | 197 | 0.59 | 0.72±0.15 |
|  |  | GA | 118 | 0.35 | 0.68±0.15 |
|  |  | GG | 20 | 0.06 | 0.66±0.09 |
|  | rs1498373 | CC | 146 | 0.43 | 0.68±0.14 |
|  |  | TC | 162 | 0.48 | 0.71±0.14 |
|  |  | TT | 30 | 0.09 | 0.75±0.18 |
|  | rs233130 | AA | 146 | 0.44 | 0.68±0.14 |
|  |  | GA | 158 | 0.47 | 0.72±0.14 |
|  |  | GG | 31 | 0.09 | 0.76±0.18 |
|  | rs233080 | GG | 166 | 0.50 | 0.71±0.15 |
|  |  | AG | 145 | 0.43 | 0.7±0.14 |
|  |  | AA | 24 | 0.07 | 0.73±0.18 |
|  | rs2474123 | GG | 111 | 0.33 | 0.67±0.14 |
|  |  | AG | 168 | 0.50 | 0.71±0.14 |
|  |  | AA | 56 | 0.17 | 0.74±0.16 |
|  | rs986639 | GG | 244 | 0.73 | 0.69±0.15 |
|  |  | CG | 86 | 0.26 | 0.73±0.13 |
|  |  | CC | 6 | 0.02 | 0.85±0.18 |
|  | rs12132677 | AA | 214 | 0.66 | 0.7±0.14 |
|  |  | CA | 101 | 0.31 | 0.71±0.13 |
|  |  | CC | 9 | 0.03 | 0.75±0.21 |
|  | rs7521189 | GG | 98 | 0.29 | 0.73±0.15 |
|  |  | AG | 154 | 0.46 | 0.72±0.15 |
|  |  | AA | 82 | 0.25 | 0.65±0.12 |
|  | rs17388437 | TT | 313 | 0.93 | 0.7±0.15 |
|  |  | CT | 24 | 0.07 | 0.71±0.15 |
|  |  | CC | 1 | 0.00 | 0.89±0 |
|  | rs11161614 | TT | 197 | 0.60 | 0.69±0.14 |
|  |  | GT | 122 | 0.37 | 0.72±0.14 |
|  |  | GG | 12 | 0.04 | 0.74±0.14 |
|  | rs553257 | TT | 215 | 0.65 | 0.71±0.15 |
|  |  | CT | 107 | 0.32 | 0.7±0.15 |
|  |  | CC | 10 | 0.03 | 0.65±0.1 |
|  | rs669173 | TT | 107 | 0.32 | 0.74±0.15 |
|  |  | CT | 161 | 0.48 | 0.71±0.15 |
|  |  | CC | 68 | 0.20 | 0.64±0.1 |
|  | rs539714 | TT | 241 | 0.72 | 0.7±0.14 |
|  |  | CT | 93 | 0.28 | 0.71±0.15 |
|  |  | CC | 2 | 0.01 | 0.91±0.03 |
|  | rs11161618 | CC | 103 | 0.30 | 0.72±0.15 |
|  |  | TC | 173 | 0.51 | 0.7±0.15 |
|  |  | TT | 63 | 0.19 | 0.69±0.13 |
|  | rs2935 | GG | 279 | 0.83 | 0.7±0.14 |
|  |  | AG | 56 | 0.17 | 0.73±0.14 |
|  |  | AA | 3 | 0.01 | 0.92±0.16 |
|  | rs13373844 | AA | 176 | 0.52 | 0.73±0.14 |
|  |  | CA | 132 | 0.39 | 0.68±0.15 |
|  |  | CC | 28 | 0.08 | 0.66±0.1 |
|  | rs3738111 | TT | 275 | 0.82 | 0.7±0.14 |
|  |  | CT | 55 | 0.16 | 0.72±0.16 |
|  |  | CC | 5 | 0.01 | 0.65±0.13 |
|  | rs877041 | GG | 118 | 0.35 | 0.72±0.15 |
|  |  | AG | 176 | 0.52 | 0.7±0.14 |
|  |  | AA | 42 | 0.13 | 0.7±0.15 |
|  | rs12568675 | TT | 273 | 0.82 | 0.7±0.14 |
|  |  | CT | 55 | 0.17 | 0.73±0.13 |
|  |  | CC | 5 | 0.02 | 0.9±0.15 |
|  | rs974874 | AA | 176 | 0.52 | 0.7±0.15 |
|  |  | CA | 141 | 0.42 | 0.71±0.14 |
|  |  | CC | 22 | 0.06 | 0.73±0.17 |
|  | rs480414 | GG | 158 | 0.47 | 0.71±0.14 |
|  |  | AG | 152 | 0.45 | 0.7±0.14 |
|  |  | AA | 27 | 0.08 | 0.7±0.17 |
|  | rs1241321 | TT | 155 | 0.46 | 0.69±0.14 |
|  |  | CT | 148 | 0.44 | 0.71±0.15 |
|  |  | CC | 33 | 0.10 | 0.73±0.15 |
|  | rs587843 | GG | 148 | 0.44 | 0.7±0.14 |
|  |  | CG | 158 | 0.47 | 0.71±0.15 |
|  |  | CC | 30 | 0.09 | 0.71±0.14 |
|  | rs10782551 | GG | 279 | 0.83 | 0.71±0.15 |
|  |  | AG | 53 | 0.16 | 0.67±0.14 |
|  |  | AA | 3 | 0.01 | 0.73±0.1 |
|  | rs1403955 | AA | 131 | 0.39 | 0.69±0.15 |
|  |  | CA | 165 | 0.49 | 0.71±0.13 |
|  |  | CC | 42 | 0.12 | 0.72±0.18 |
|  | rs1403951 | GG | 96 | 0.28 | 0.69±0.15 |
|  |  | TG | 174 | 0.52 | 0.71±0.14 |
|  |  | TT | 67 | 0.20 | 0.71±0.16 |
| DDAH2 | rs805287 | TT | 163 | 0.48 | 0.7±0.16 |
|  |  | CT | 142 | 0.42 | 0.71±0.14 |
|  |  | CC | 32 | 0.09 | 0.71±0.12 |
|  | rs6916278 | GG | 291 | 0.87 | 0.71±0.15 |
|  |  | AG | 38 | 0.11 | 0.71±0.14 |
|  |  | AA | 4 | 0.01 | 0.69±0.09 |
|  | rs805285 | CC | 186 | 0.55 | 0.7±0.15 |
|  |  | GC | 125 | 0.37 | 0.7±0.14 |
|  |  | GG | 27 | 0.08 | 0.73±0.12 |
|  | rs15574 | CC | 216 | 0.64 | 0.71±0.15 |
|  |  | TC | 102 | 0.30 | 0.7±0.13 |
|  |  | TT | 17 | 0.05 | 0.75±0.14 |
|  | rs805294 | TT | 138 | 0.41 | 0.71±0.15 |
|  |  | CT | 147 | 0.43 | 0.7±0.14 |
|  |  | CC | 54 | 0.16 | 0.7±0.14 |
|  | rs805293 | AA | 109 | 0.32 | 0.7±0.14 |
|  |  | TA | 148 | 0.44 | 0.7±0.15 |
|  |  | TT | 79 | 0.24 | 0.71±0.14 |
|  | rs9267551 | GG | 272 | 0.80 | 0.7±0.14 |
|  |  | CG | 58 | 0.17 | 0.72±0.15 |
|  |  | CC | 8 | 0.02 | 0.71±0.21 |
|  | rs2272592 | GG | 239 | 0.71 | 0.7±0.14 |
|  |  | AG | 88 | 0.26 | 0.71±0.15 |
|  |  | AA | 10 | 0.03 | 0.74±0.17 |
|  | rs3131383 | CC | 267 | 0.80 | 0.71±0.14 |
|  |  | AC | 62 | 0.19 | 0.68±0.15 |
|  |  | AA | 6 | 0.02 | 0.59±0.17 |
|  | rs3131382 | GG | 304 | 0.90 | 0.7±0.14 |
|  |  | AG | 26 | 0.08 | 0.71±0.13 |
|  |  | AA | 6 | 0.02 | 0.74±0.24 |

S1B: Means of untransformed serum ADMA concentrations (μmol/L) by number of minor alleles of the of the two most significantly associated *DDAH1* (rs669173) and *DDAH2* (rs3131383) SNPs combined.

| No of minor alleles | N | ADMA μmol/L (mean±SD) |
| --- | --- | --- |
| 0 | 84 | 0.74±0.15 |
| 1 | 151 | 0.73±0.14 |
| 2 | 84 | 0.66±0.12 |
| 3 | 14 | 0.60±0.137 |
| 4 | 2 | 0.54±0.06 |
| Total | 335 | 0.71±0.15 |
